# Transcriptomic and metabolomic analyses reveal mechanisms of adaptation to salinity in which carbon and nitrogen metabolism is altered in sugar beet roots

**DOI:** 10.1186/s12870-020-02349-9

**Published:** 2020-04-03

**Authors:** Lei Liu, Bin Wang, Dan Liu, Chunlei Zou, Peiran Wu, Ziyang Wang, Yubo Wang, Caifeng Li

**Affiliations:** College of Agronomy, Northeast Agricultural University, Harbin, Heilongjiang China

**Keywords:** *Beta vulgaris* L., Salt tolerance, Multiomic analysis, Carbon and nitrogen metabolism

## Abstract

**Background:**

*Beta vulgaris* L. is one of the main sugar-producing crop species and is highly adaptable to saline soil. This study explored the alterations to the carbon and nitrogen metabolism mechanisms enabling the roots of sugar beet seedlings to adapt to salinity.

**Results:**

The ionome, metabolome, and transcriptome of the roots of sugar beet seedlings were evaluated after 1 day (short term) and 7 days (long term) of 300 mM Na^+^ treatment. Salt stress caused reactive oxygen species (ROS) damage and ion toxicity in the roots. Interestingly, under salt stress, the increase in the Na^+^/K^+^ ratio compared to the control ratio on day 7 was lower than that on day 1 in the roots. The transcriptomic results showed that a large number of differentially expressed genes (DEGs) were enriched in various metabolic pathways. A total of 1279 and 903 DEGs were identified on days 1 and 7, respectively, and were mapped mainly to 10 Kyoto Encyclopedia of Genes and Genomes (KEGG) pathways. Most of the genes were involved in carbon metabolism and amino acid (AA) biosynthesis. Furthermore, metabolomic analysis revealed that sucrose metabolism and the activity of the tricarboxylic acid (TCA) cycle increased in response to salt stress. After 1 day of stress, the content of sucrose decreased, whereas the content of organic acids (OAs) such as L-malic acid and 2-oxoglutaric acid increased. After 7 days of salt stress, nitrogen-containing metabolites such as AAs, betaine, melatonin, and (S)-2-aminobutyric acid increased significantly. In addition, multiomic analysis revealed that the expression of the gene encoding xanthine dehydrogenase (XDH) was upregulated and that the expression of the gene encoding allantoinase (ALN) was significantly downregulated, resulting in a large accumulation of allantoin. Correlation analysis revealed that most genes were significantly related to only allantoin and xanthosine.

**Conclusions:**

Our study demonstrated that carbon and nitrogen metabolism was altered in the roots of sugar beet plants under salt stress. Nitrogen metabolism plays a major role in the late stages of salt stress. Allantoin, which is involved in the purine metabolic pathway, may be a key regulator of sugar beet salt tolerance.

## Background

At present, 20% of arable land and nearly 50% of irrigated land have been salinized [1, 2]. Soil salinization poses an enormous threat to agricultural production, and has become a globally complex environmental problem [3]. High salt concentrations generally lead to plant ion imbalance, infiltration and oxidative damage, which can lead to wilting and plant death [4]. Therefore, improving the salt tolerance of crops has become an important research topic.

Owing to its excellent salt tolerance, sugar beet is used as a model sugar crop species for studying the salt tolerance mechanism of plants [5–8]. Compared with other plants species, sugar beet can better withstand high salt stress and drought stress [9]. Plants respond to salt stress by accumulating osmotic regulators, selectively absorbing salt ions, partitioning salt ions, and enhancing their antioxidant capability [10, 11].

At present, it is known that the salt tolerance mechanism of plants is related to the accumulation of primary metabolites in plant tissues, such as sugars, amino acids (AAs), polyols, organic acids (OAs) and hormones [12, 13]. In sugar beet, secondary metabolites, such as betaine, can protect membranes and can enhance the activity of antioxidant enzymes; as such, these metabolites can promote the elimination of intracellular reactive oxygen species (ROS) and play an active role preventing lipid peroxidation [14]. The carbon- and/or nitrogen-containing compounds in sugar beet can improve overall plant adaptability under unfavorable growth conditions [15–17]. It is well known that nitrogen and carbohydrates are important factors limiting crop growth, but it is not known how these two metabolic processes are rebalanced when plants are stressed. Although there have been numerous studies of the response mechanism of sugar beet to salt stress, most of these investigations have been limited to the ecophysiological level or restricted to a single pathway. Knowledge concerning the physiological and molecular mechanisms occurring in sugar beet under salt stress isincomplete. Moreover, there is little information available regarding the relationship between metabolomic responses and transcriptomic responses to salt stress in sugar beet, particularly in the roots; most related studies have focused on the shoots or leaves of plants.

Transcriptomic analyses of cotton (*Gossypium* spp.), soybean (*Glycine max* (Linn.) Merr.), alfalfa (*Medicago sativa* L.), barley and other plant species have revealed a large number of DEGs related to secondary metabolites, hormone synthesis, nitrogen absorption, ROS clearance, cell membrane stability, and signal transduction pathways under salt stress [18–21]. However, few studies have investigated the changes in carbon and nitrogen metabolism in plants as a whole. Some studies have shown that the content of TCA-related OAs in sugar beet leaves increases under salt stress and that proline, mannitol and putrescine help sugar beet leaves adapt to salt stress [22, 23]. However, how sugar beet regulates carbon and nitrogen metabolism under salt stress and whether this species has a unique salt tolerance regulatory pathway remain unknown. However, all metabolic changes are controlled by genes, so additional research on changes in gene expression is needed.

Roots, organs that directly encounter salt stress, act as a reservoir of carbohydrates [24, 25]. Therefore, the salt tolerance of roots is closely related to plant growth. By combining transcriptomic and metabolomic approaches, weelucidated the aforementioned protective mechanisms to better understand how plants regulate carbon and nitrogen metabolism to adapt to salt stress. The purpose of this study was to determine how sugar beet roots ensure the balance between carbon metabolism and nitrogen metabolism in response to salt stress, to determine the pathways important to sugar beet root adaptation and tolerance to salt stress, and to identify key genes and metabolites involved in the salt stress response. Therefore, there is great theoretical and practical value in performing physiological research on sugar beet salt stress and in revealing the mechanisms governing salt tolerance.

## Results

### Physiological changes in sugar beet under salinity stress

**Fig. 1:**
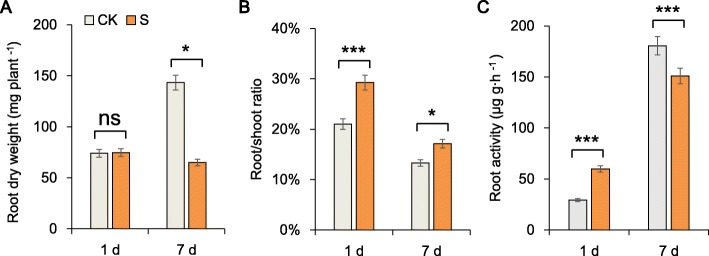
Plant growth in response to salt stress (300 mM Na^+^) or control (no salt) for 1 day and 7 days. **a** Root dry weight (DW), **b** root/shoot ratio and (**c**) root activity (RA) were measured in sugar beet seedlings. The data shown are the means of three biological replicates (± SD). S, salt stress; CK, control. Significant differences are indicated (ns, not significant; *,*P* < 0.05; ***,*P* < 0.001)

Our preliminary experiments showed that sugar beet plants could complete the vegetative growth phase in a solution whose maximum salt concentration was 300 mM (NaCl+Na_2_SO_4_). The root growth of sugar beet plants under salt stress was compared after 1 day and 7 days of the stress. The root biomass of sugar beet plants under salt stress was significantly (54.65%) lower than that in the plants under the control (CK) conditions (Fig. 1a). This result indicated that sugar beet suffers from severe salt stress, reflected by inhibited root growth. However, the root activity (RA) of plants under salt stress was 104.04% greater than that in plants under the CK conditions after 1 day of stress; the RA was indicated by the activity of succinate dehydrogenase in the mitochondria of living cells. This association was verified by the significant increase in malic acid, which is presented below. Moreover, the root/shoot ratio under stress was 8.26 and 3.83% greater than that under the CK conditions on days 1 and 7, respectively (Fig. 1b, c). In this paper, the sugar beet growth slowed, the root metabolism increased, and the activity of the tricarboxylic acid (TCA) cycle increased in response salt stress.

**Fig. 2:**
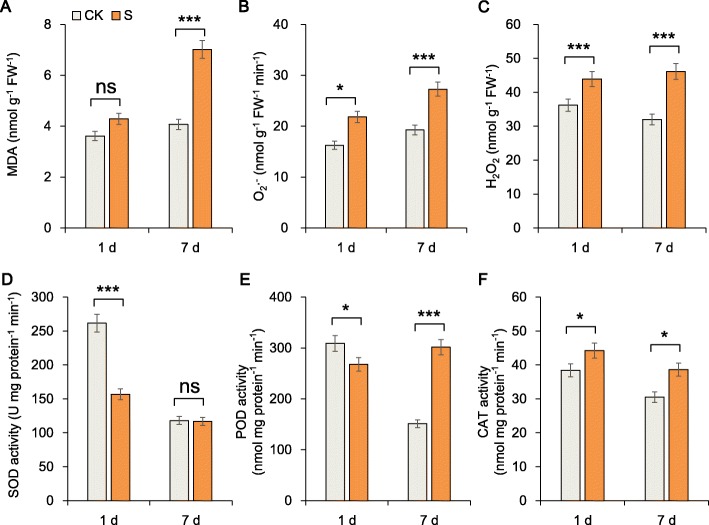
Root physiological changes in response to salt stress for 1 day and 7 days. **a** Malondialdehyde (MDA) content, **b** superoxide radical (O_2_·^−^) content, **c** hydrogen peroxide (H_2_O_2_) content, **d** superoxide dismutase (SOD), **e** peroxidase (POD) activity and **f** catalase (CAT) activity were measured in sugar beet seedlings. The data shown are the means of three biological replicates (± SD). Significant differences are indicated (ns, not significant; *,*P* < 0.05; ***,*P* < 0.001)

**Fig. 3:**
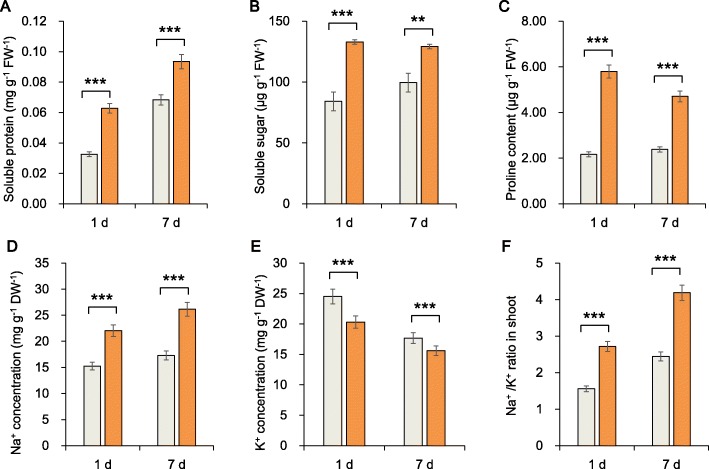
Root growth in response to salt stress for 1 day and 7 days. **a** Soluble protein content, **b** soluble sugar content, **c** proline content, **d** Na^+^ concentration, **e** K^+^ concentration and **f** Na^+^/K^+^ ratio were measured in sugar beet seedlings. The data shown are the means of three biological replicates (± SD). Significant differences are indicated (ns, not significant; **,*P* < 0.01; ***,*P* < 0.001)

We studied the ROS and oxidative defense system of the sugar beet roots and found that the ROS content in roots increased over time (Fig. 2a, b, c). The contents of malondialdehyde (MDA), superoxide anion (O_2_**·**^−^) and hydrogen peroxide (H_2_O_2_) after 7 days of salt treatment were greater than those after 1 day of stress. However, the protective enzyme activity increased: peroxidase (POD) activity in the plants under salt stress conditions was 2 times greater than that in the plants under the CK conditions on day 7. This result suggests that POD may play a key role in salt tolerance, which we verified at the molecular level (Fig. 2d, e, f). Further, the soluble protein content, soluble sugar content and proline content increased significantly after 1 and 7 days of salt stress (Fig. 3a, b, c).

Compared with that in the CK plants, the Na^+^ content in the roots of plants after 1 and 7 days increased significantly by 44.59 and 51.31%, and the K^+^ content decreased significantly by 17.14 and 11.60%%, respectively. Compared with the respective control, the increase in the Na^+^/K^+^ ratio under salt stress on day 7 (71.17%) was lower than that on day 1 (74.59%) (Fig. 3d, e, f). In this high-salt environment, the levels of ROS and toxic ions in the plants were still too high. Most of these osmotic regulatory substances are involved in the stress response; thus, we believe that important regulatory substances are also involved in the salt tolerance of sugar beet roots. Therefore, we performed a metabolic analysis, which is described below.

### Assessment of metabolic changes in sugar beet during salinity acclimation

To monitor the metabolic acclimation processes of sugar beet under salt stress, ultra-performance liquid chromatography-mass spectrometry (UPLC-MS) was performed to identify differentially expressed metabolites. In total, 127 differentially expressed metabolites (24 AAs, 21 OAs, 9 amines, 9 carbohydrates, 11 lipids, 9 alkaloids, 7 nucleic acids, 4 vitamins, 2 cofactors, 2 sugar alcohols, 2 hormones and 28 other compounds) were reproducibly identified in the roots of plants under the CK conditions and salt stress conditions (Table S1).

**Fig. 4:**
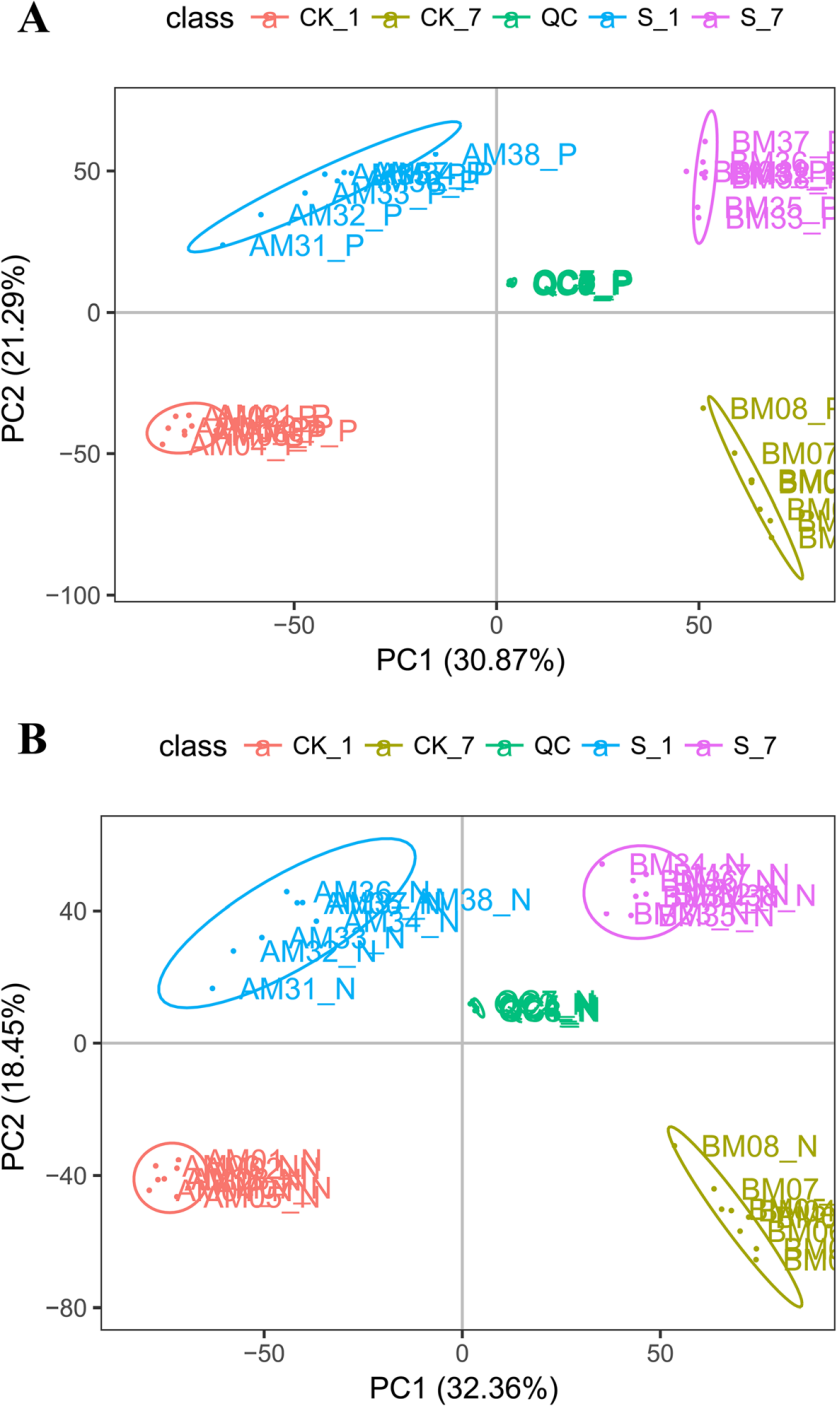
Root metabolome variation among samples as determined by principal component analysis (PCA). **a** Positive electrospray ionization (ESI) mode, **b** negative ESI mode; PC1 represents the first principal component; PC2 represents the second principal component. CK_1, control on day 1; CK_7, control on day 7; S_1, salt on day 1; S_7, salt on day 1; QC, quality control (a mixture of experimental samples prepared in equal amounts). The data shown are the means of eight biological replicates

According to principal component (PC) analysis, in positive electrospray ionization (ESI) mode, the first PC (PC1) explained 30.87% of the total measured metabolite variation in the direction of the treatments, while the second PC (PC2) accounted for 21.29% of the total variation, which was attributed partially to the treatment differences and partially to the temporal differences under salt stress. Similar results were obtained in negative ESI mode (Fig. 4).

**Fig. 5:**
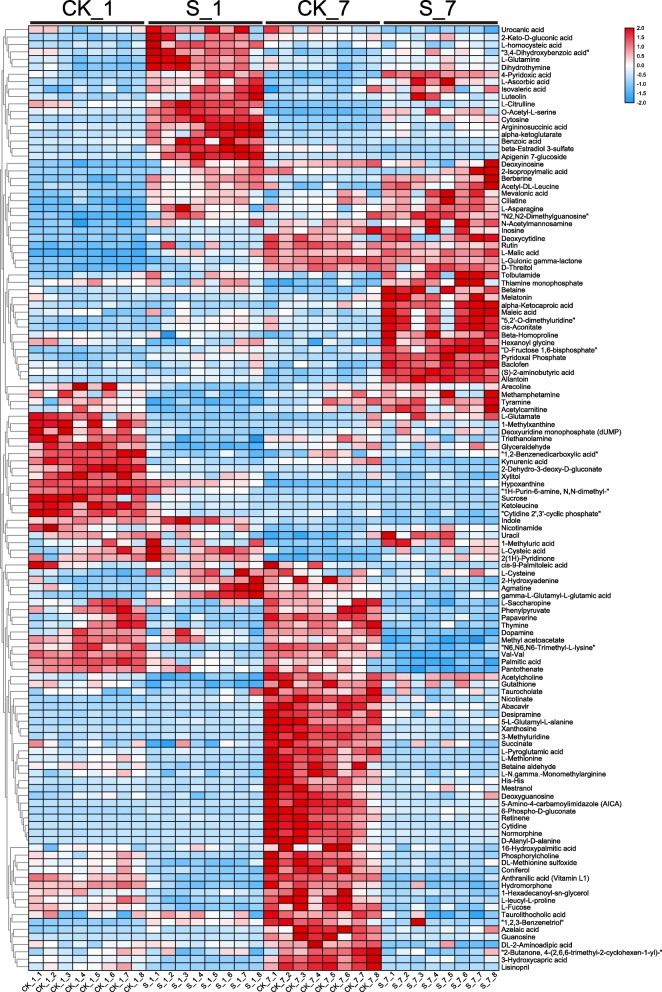
Results of hierarchical clustering analysis of altered metabolite pools in sugar beet subjected to salt stress (*P* < 0.01). Heatmap color indicate the abundance of each metabolite under salt stress on day 1 and 7

On the basis of a heat map analysis (Fig. 5) and the data in Table S2, we deduced that the abundance of the primary metabolites was significantly affected after exposure to salt stress for 1 day (40 increased in abundance, and 33 decreased) and 7 days (30 increased and 55 decreased). Compared with those under the CK conditions, most AAs under salt stress conditions were significantly enriched on day 1, including L-glutamine and L-asparagine. Similarly, most of the OAs, such as cis-aconitate, benzoic acid L-malic acid, and alpha-ketoglutarate, were significantly enriched under salt stress on both days; however, the abundance of all lipids decreased on day 7.

Compared with the CK plants, the salt-stressed plants presented the most obvious changes 2-isopropylmalic acid (27.4-fold on day 1 but no obvious differences on day 7). Nitrogen-containing metabolites such as allantoin, betaine, L-citrulline and two hormones (melatonin and (S)-2-aminobutyric acid) increased significantly under salt stress conditions compared with the CK conditions only on day 7. The allantoin content presented a larger fold change (6.93-fold) than did the betaine content (6.16-fold).

### Transcriptomic analysis of sugar beet under salinity stress

The Illumina HiSeq 4000 was utilized for generating the reference transcriptome for *Beta vulgaris* L. in the case of salt stress. Table 1 displays the 48,862,905, 51,492,828, 51,606,390, and 53,096,544 total raw reads acquired based on CK and salt-stressed plants. Following the removal of the low-quality sequences, 48,122,135, 50,581,785, 50,804,895 and 52,238,759 valid reads were preserved in subsequent assembly. The GC percentages were 42.00, 42.50, 42.17, and 42.33%, respectively, while the Q20 values were over 99.56% at the sequencing error rate of < 1%. According to the above findings, those sequencing data were in high quality with great quantity, which guaranteed the accuracy of sequence assembly, as well as sufficient coverage of the transcriptome. On the basis of comparisons of 18 randomly selected genes between the salt stress and CK conditions, the quantitative real-time PCR (qRT-PCR) results showed good agreement with the RNA sequencing (RNA-seq) data (Table S3).

**Table 1:** Summary statistics of the sequencing results. CK_1, control on day 1; CK_7, control on day 7; S_1, salt on day 1; S_7, salt on day 1

Sample	Raw Data (reads)	Valid Data (reads)	Valid Ratio %	Q20%	Q30%	GC content %
CK_1	48,862,905	48,122,135	98.48	99.65	95.63	42.00
S_1	51,492,828	50,581,785	98.22	99.65	95.73	42.50
CK_7	51,606,390	50,804,895	98.45	99.71	96.20	42.17
S_7	53,096,544	52,238,759	98.38	99.65	95.78	42.33

### Functional analysis of DEGs

**Fig. 6:**
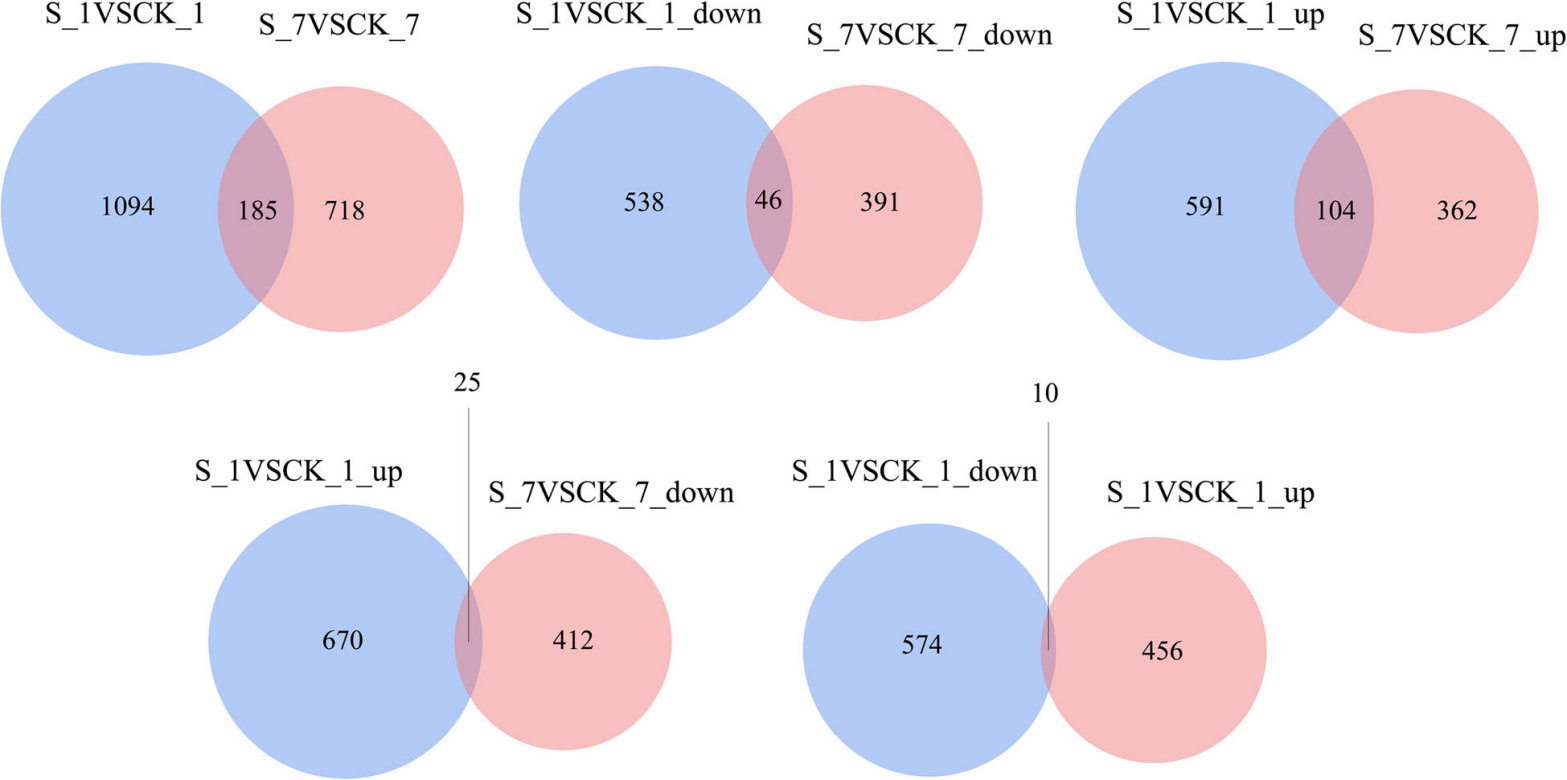
Global comparison of differentially expressed genes (DEGs) in the roots of sugar beet at different stages of salt stress. **a** Distribution of all DEGs, **b** downregulated DEGs and **c** upregulated DEGs annotated in samples treated with salt for 1 day (blue circles) and 7 days (red circles); **d** upregulated DEGs on day 1 and downregulated DEGs on day 7; **e** downregulated DEGs on day 1 and upregulated DEGs on day 7

**Fig. 7:**
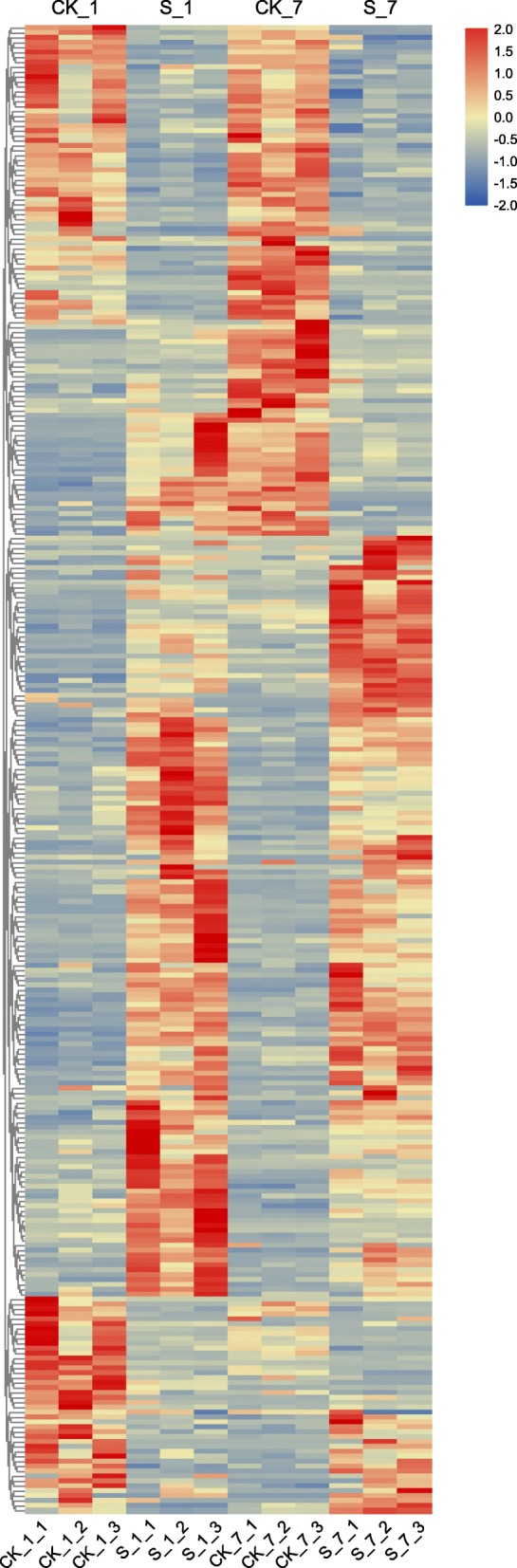
Cluster analysis of coexpressed transcripts at different stages of salt stress. The heat map color indicates the abundance of each transcript at different stages of salt stress (*P* < 0.05). The heatmap presents normalized FPKM expression values. The blue color indicates low-intensity spots, while the red color indicates spots with high signal

Of the 1279 and 903 differentially expressed genes (DEGs) identified after 1 and 7 days of stress in sugar beet, respectively (Fig. 6), the expression levels of 104 and 46 genes in the roots were up- or downregulated under salt stress both on both days. However, the expression of 25 DEGs was first upregulated but then downregulated, while that of 10 DEGs was first downregulated and then upregulated, indicating that the transcriptomic responses of sugar beet to salt stress were largely timespecific. Subsequently, hierarchical clustering analysis was carried out for obtaining the comprehensive transcription profiles for those coexpressed transcripts at various salt stress stages (Fig. 7). The clustering profiles indicated that salt stress significantly affected the transcriptional profiles of the coexpressed transcripts. The number of upregulated genes in the salt stress treatment compared with the CK treatment was greater than the number of downregulated genes among these coexpressed transcripts. Moreover, there were more downregulated genes after 7 days of salt stress than after 1 day of salt stress.

According to Gene Ontology (GO) analysis (Table S4), the DEGs (*P* value < 0.05) on day 1 (369, 483 and 610) and day 7 (289, 46 and 420) were annotated with three GO functions: Biological process, Cellular component and Molecular function, respectively. On day 1, “oxidation-reduction process” (GO:0055114, 98 genes), “metabolic process” (GO:0008152, 50 genes) and “transmembrane transport” (GO:0055085, 37 genes) were the three most enriched GO terms in the Biological process ontology. On the 7th day, the “redox process” (*n* = 78 genes), the “metabolic process” (*n* = 43), together with the “carbohydrate metabolic process” (GO:0005975, *n* = 27), accounted for the top three most significantly enriched gene ontology (GO) terms in Biological Process category. On the 1st and 7th days, the “metal ion binding” (GO:0046872, *n* = 92), the “oxidoreductase activity” (GO:0016491, *n* = 82), as well as the “hydrolase activity” (GO:0016787, *n* = 57) represented the top three most significantly enriched GO terms in Molecular Function category.

**Fig. 8:**
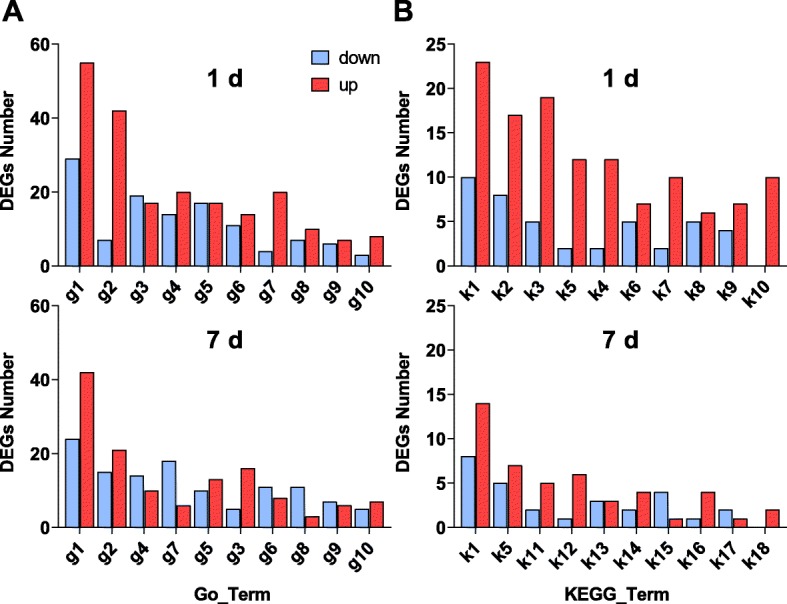
**a** Plots of the top 10 Gene Ontology (GO) terms from the biological process category of genes, **b** plots of the top 10 Kyoto Encyclopedia of Genes and Genomes (KEGG) pathways (S_1 vs CK_1 and S_7 vs CK_7). g1, Oxidation-reduction process; g2, Metabolic process; g3, Transmembrane transport; g4, Regulation of transcription, DNA-templated; g5, Transport; g6, Transcription, DNA-templated; g7, Carbohydrate metabolic process; g8, Protein phosphorylation; g9, Response to oxidative stress; g10, Proteolysis; k1, Phenylpropanoid biosynthesis; k2, Carbon metabolism; k3, Biosynthesis of amino acids; k5, Starch and sucrose metabolism; k4, Glycolysis/Gluconeogenesis; k6, Carbon fixation in photosynthetic organisms; k7, Cysteine and methionine metabolism; k8, beta-Alanine metabolism; k9, Glycine, serine and threonine metabolism; k10, Fatty acid metabolism; k11, alpha-Linolenic acid metabolism; k12, Arginine and proline metabolism; k13, Carotenoid biosynthesis; k14, Valine, leucine and isoleucine degradation; k15, Nitrogen metabolism; k16, Alanine, aspartate and glutamate metabolism; k17, Nicotinate and nicotinamide metabolism; k18, Tyrosine metabolism

The DEGs were mapped to 10 GO terms (on both days) from the biological process category (Fig. 8a). The most abundant DEGs were classified as being associated with oxidation-reduction processes (55 up- and 29 downregulated genes), followed by metabolic processes (42 up- and 7 downregulated genes) and transmembrane transport (17 up- and 19 downregulated genes) on day 1. Only 141 upregulated genes were enriched on day 7, resulting in a nearly 0.5-fold decrease compared with the value after 1 day of salt stress. Additionally, there was a sharp increase in the expression of genes related to metabolic processes on day 1. Moreover, most of the DEGs were enriched in oxidation-reduction or metabolic processes, and there were more upregulated genes than downregulated genes on both days. However, the regulation of the carbohydrate metabolic process was more enriched among the downregulated DEGs than the upregulated DEGs on day 7. These findings indicate that salt stress can severely affect the metabolism and oxidation-reduction process in plants.

We further identified KEGG Orthology terms (*P* value < 0.05) (Table S5). In total, 273 and 78 DEGs were located in 28 and 13 KEGG pathways on day 1 and 7, respectively. Metabolic pathways including phenylpropanoid biosynthesis (33 genes), carbon metabolism (25 genes) and amino acid biosynthesis (24 genes) in sugar beet were influenced drastically by salt treatment on day 1. On day 7, the main metabolic pathways significantly affected by salt stress included phenylpropane biosynthesis (22 genes), starch and sucrose metabolism (12 genes) and alpha-linolenic acid metabolism (8 genes).

Figure 8a shows the top 10 pathways with significant differences (*P* value < 0.05). The number of genes associated with the KEGG pathways was lower on day 7 than on day 1. The pathways contained more upregulated DEGs than downregulated DEGs under salt stress, especially on day 1. A total of 33 unigenes (23 upregulated, 10 downregulated) that mapped to phenylpropanoid biosynthesis were affected on day 1, and 22 unigenes (14 upregulated, 8 downregulated) were affected on day 7 in response to salt stress. These changes were followed by changes in genes related to carbohydrate metabolism, starch and sucrose metabolism and AA biosynthesis, which were also affected at both stages of salt stress. The biological functions of the unigenes annotated in the metabolism category consist of the catalysis of metabolic processes or generation of energy for primary and secondary metabolite production under salt stress. The number of genes associated with the KEGG pathways decreased on day 7 compared with day 1.

### Gene expression in response to salinity

On the basis of criteria that included a fold change greater than 2 and a significance (*P* value) greater than 0.05 determined via t tests, we selected highly DEGs (Table S6). These genes could be classified into five major types on the basis of their differential responses to salt treatment after 1 day and 7 days. Type I (high expression only on day 1) included 12 DEGs whose expression significantly increased in the roots in response to salt stress on day 1 but whose expression was not significantly different on day 7. Of the type I genes, the expression of ethylene-responsive transcription factor 2 (LOC104892796) increased most dramatically (> 221-fold) on day 1. Type II (high expression only on day 7) included 12 DEGs whose expression significantly increased in the roots in response to salt stress only on day 1. Of these type II genes, the expression of protein P21-like (LOC104892315) increased most dramatically (96-fold), and the expression of ethylene-responsive transcription factor 4 (LOC104900638) increased by > 45-fold on day 7.

Type III (high expression on both days 1 and 7) included 104 DEGs whose expression significantly increased in the roots in response to salt on both day 1 and day 7. We found that three of the common highly DEGs encoded a late embryogenesis abundant (LEA) protein. Interestingly, three genes were associated with enhanced cation toxicity and osmotic stress tolerance in the sugar beet seedlings. We also found that expansin-like B1 was governed by three genes and two genes encoding cytochrome on days 1 and 7, respectively. However, 32 genes lacked functional annotation. In total, there were 38 enzyme-encoding genes in this list, including one gene encoding 2-hydroxyflavanone dehydratase, one gene encoding one cationic POD, two genes encoding protein phosphatase, one gene encoding ureide permease 2, one gene encoding GDSL esterase/lipase (At5g55050), one gene encoding alpha-1,4-glucan-protein synthase and two genes encoding protein phosphatase*.* Additionally, one gene was annotated as spermine synthase. Other genes were also identified, including one gene encoding polygalacturonase, one gene encoding auxin-responsive protein IAA29, one gene encoding dirigent protein 21 and one gene encoding chloroplastic choline monooxygenase.

Type IV included 25 DEGs whose expression increased significantly in the roots in response to salt stress on day 1 but whose expression decreased on day 7, including one gene encoding the ethylene-responsive transcription factor 3, one gene encoding POD 11, one gene encoding POD 20, one gene encoding GDSL esterase/lipase and one gene encoding the BAG family molecular chaperone regulator 6. Type V included 10 DEGs whose expression decreased significantly in the roots in response to salt stress on day 1 but whose expression increased on day 7, including one gene encoding the ethylene-responsive transcription factor ERF107 and one gene encoding the auxin-binding protein ABP19a.

### Integrative analysis of gene expression and metabolic changes under salt stress conditions

**Fig. 9:**
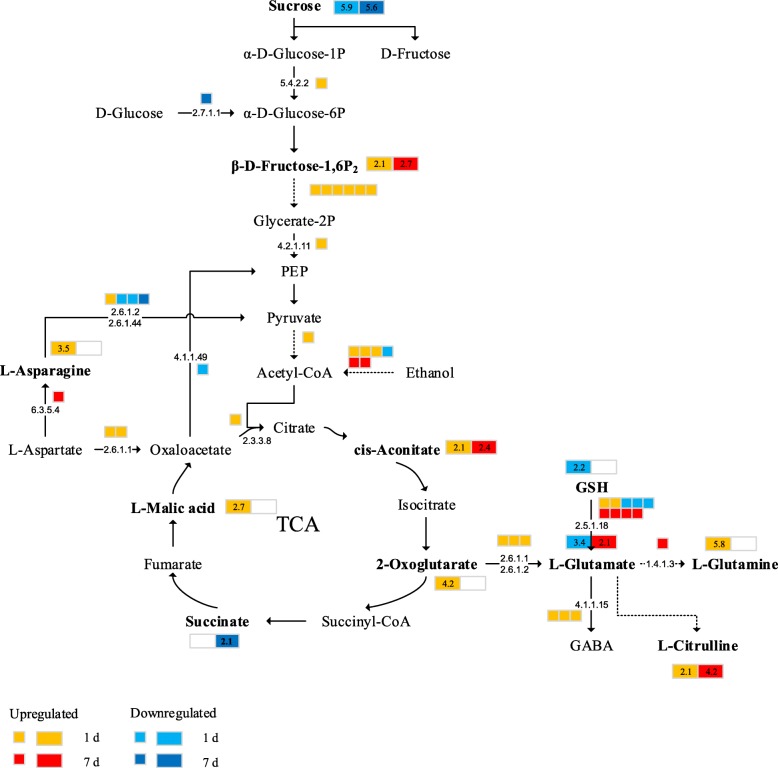
Pathway analysis of sugar beet under salt stress. The proposed metabolic pathways are based on the Kyoto Encyclopedia of Genes and Genomes (KEGG) database of metabolic pathways. The metabolites, written in bold, were detected in this study. Omitted metabolic processes are represented by dashed lines. The small icons represent the genes, while the large icons represent the metabolites. The orange and red colors indicate upregulated expression on days 1 and 7, while the light blue and dark blue colors indicates downregulated expression on days 1 and 7, respectively

We identified a relationship of major gene expression with the metabolic product levels. Table S7 lists these metabolites, related genes, as well as the major metabolic pathways. According to our results, salt stress had significant influence on these pathways. Those primary pathways involved in TCA cycle, glycolysis, metabolism of the aspartate family and glutamate biosynthesis are presented in the metabolism overview. As shown in Fig. 9, the expression of 10 genes encoding glycolysis enzymes, such as phosphoglucomutase [EC 5.4.2.2], enolase [EC 4.2.1.11] and pyruvate decarboxylase [EC 4.1.1.1], was upregulated in the plants under salt stress conditions for 1 day compared with the plants under the CK conditions. In the TCA cycle, the expression of the gene encoding the citrate cleavage enzyme [EC 2.3.3.8] was upregulated after day 1 of stress. Furthermore, the glycolysis and sucrose metabolic pathways become enhanced, the sucrose content decreases, and a large amount of OAs and AAs such as cis-aconitate, L-malic acid, succinate, alpha-ketoglutarate, L-asparagine, and L-glutamine are produced.

These changes in the expression of these glycolysis genes may have increased the contents of cis-aconitate, 2-oxoglutarate and L-malic acid by 2.1-, 4.2- and 2.7-fold, respectively, after 1 day of salt stress. Compared with that in the plants under the CK conditions, the cis-aconitate content in the plants under salt stress conditions increased 2.4-fold on day 7. The expression of the genes encoding aspartate aminotransferase [EC 2.6.1.2] and alanine transaminase [EC 2.6.1.1] was upregulated, causing 2-oxoglutarate to generate L-glutamate, after 1 day of salt stress, whereas the contents of L-glutamate and GSH decreased. At the same time, the expression of three genes encoding glutamate decarboxylase [EC 4.1.1.15] was upregulated, and the downstream products of L-glutamine and citrulline were more abundant, resulting in glutamate shortage under stress. The regulation of glycolysis and the TCA cycle indicates that the changes in the abundance of ATP probably occurred at the early stage of salt stress. These ATP molecules in turn provide energy for other physiological functions.

**Fig. 10:**
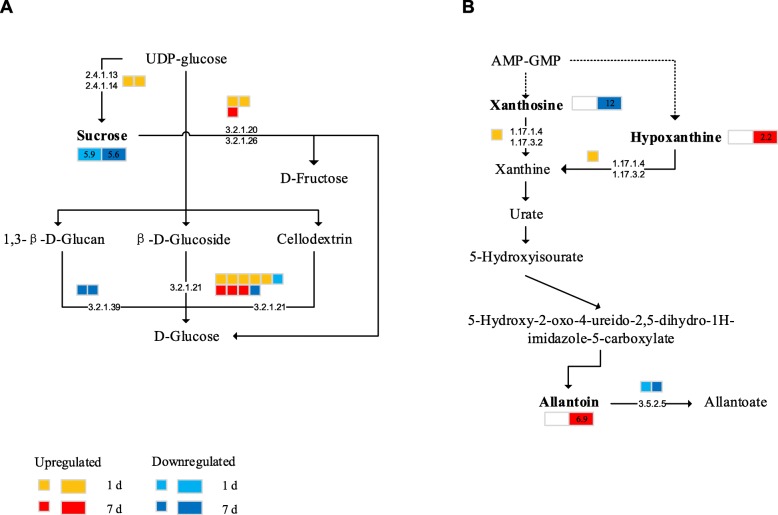
Altered expression of genes involved in (**a**) sucrose metabolism and (**b**) purine metabolism. The metabolites, written in bold, were detected in this study. Omitted metabolic processes are represented by dashed lines. The small icons represent the genes, while the large icons represent the metabolites. The orange and red colors indicate upregulated expression on days 1 and 7, while the light blue and dark blue colors indicate downregulated expression on days 1 and 7, respectively

The expression of most genes associated with sucrose metabolism was upregulated under salt stress (Fig. 10a), including the gene encoding sucrose synthase (SS [EC 2.4.1.13]). However, sucrose was lower in the plants under salt stress than in plants under the CK conditions. The expression of the invertase (INV [EC 3.2.1.26]) gene and the beta-glucosidase gene was upregulated under salt stress, which might be responsible for the observed increase in soluble sugar. These changes in the expression of key genes could alter sucrose metabolism in plants. After 7 days of salt stress, glutathione S-transferase [EC 2.5.1.18] catalyzed the production of glutamate which led to the accumulation of more citrulline. Interestingly, the content of allantoinase (ALN), which is involved in the purine metabolic pathway, was significantly downregulated, resulting in a large accumulation of allantoin on day 7 (Fig. 10b). Urea metabolism in sugar beet begins with the production of xanthosine from adenosine monophosphate (AMP) and guanosine monophosphate (GMP) by deamination, followed by the hydrolysis of xanthine and hypoxanthine by xanthine dehydrogenase (XDH [EC 1.17.1.4]). Purine xanthine is subsequently oxidized to urate by XDH. Uricase (uric acid oxidase, UO; EC 1.7.3.3) converts uric acid to 5-hydroxyisocyanate (5-HIU), and 5-HIU is metabolized to urine by the 2-oxo-4-hydroxy-4-carboxy-5-cyanosylurea-imidazoline (OHCU) intermediate. ALN [E.C. 5.5.2.5] subsequently catalyzes the breakdown of allantoin to allantoic acid. In this study, the expression of the gene encoding XDH increased, which led to the breakdown of xanthine to form urate. At the same time, the expression of the ALN gene was downregulated, inhibiting the breakdown of allantoin, which began to accumulate on the first day of stress. This suggests that the accumulation of allantoin may increase the adaptability of sugar beets to salt stress.

**Fig. 11:**
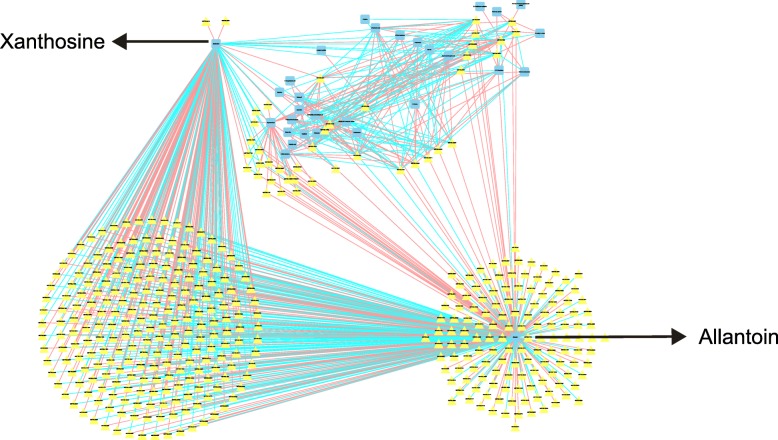
Pearson correlation analysis of differential genes and metabolites under all salt stress on day 7 (r > 0.5, *P* < 0.05). Squares represent metabolites, and triangles represent genes. The same type of metabolite or gene is marked with the same color. The connection between genes and metabolites represents a correlation between the two, with red representing a positive correlation and blue representing a negative correlation

**Fig. 12:**
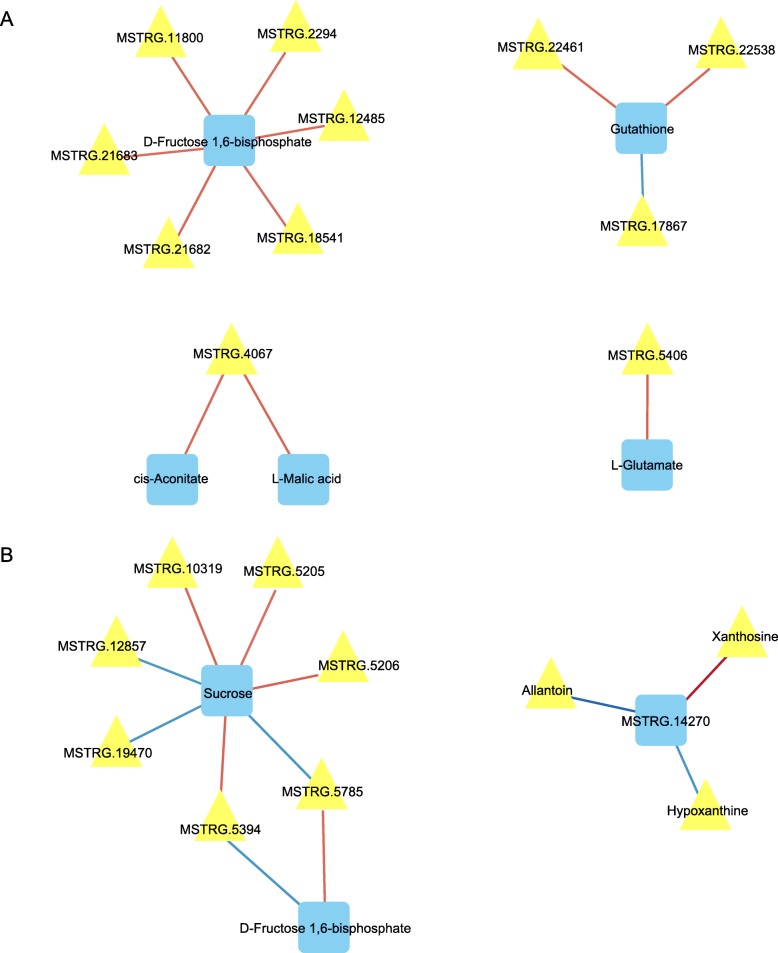
Association of the major genes of metabolic pathways and metabolites on day 1 (**a**) and day 7 (**b**). See Fig. 11 for more details

We constructed a network diagram of the relevant genes and metabolites (Figs. 11 and 12). We first analyzed the correlations of all metabolites and genes on day 7 of salt stress (r > 0.5, *P* < 0.05). The results showed that the majority of genes were associated with only xanthosine and allantoin in the purine metabolic pathway. We subsequently performed a correlation analysis of the genes and metabolites in each pathway (r > 0.8, *P* < 0.001) (Fig. 12a and b). The results showed that the changes in sucrose and fructose-1,6-diphosphate were significantly correlated with several genes, but the related genes were different between the early and late stages. MSTRG.14270 (*allantoinase*) was the main gene involved in the salt stress response; this gene was significantly positively correlated with xanthine and significantly negatively correlated with allantoin and hypoxanthine in purine metabolism.

## Discussion

### Osmotic stress, ion toxicity and salt tolerance of roots

High salt concentrations reduce the water potential of an environment, leading to osmotic shock, and excess sodium ions inhibit cellular metabolic processes and produce secondary metabolites [26]. Moreover, most of the genes involved in redox processes are expressed in abundance. Our results also show that, under salt stress, multiple sugar beet genes encoding LEA (3) POD (2) proteins are upregulated. LEA proteins are involved in dehydration tolerance, and they are often known for their late accumulation during seed development and their role as osmoprotectants, membrane stabilizers, antioxidants and chaperones to protect plants from abiotic stress [27, 28]. There is increasing evidence that LEA proteins play a role in a range of biological processes, including plant growth and development, morphogenesis and senescence [29–32]. Huang et al. [29] reported that LEA proteins can enhance the expression and enzyme activity of NADPH oxidase and of antioxidant defense enzymes such ascorbic acid peroxidase (APX), catalase (CAT) and superoxide dismutase (SOD) to alleviate oxidative damage in rice. Beet varieties with stronger salt tolerance have higher levels of protective enzyme activities (such as SOD and APX activities) [33].

Generally, ion toxicity associated with salt stress is mainly caused by Na^+^. An increase in the Na^+^ concentration in plants during salt stress has been confirmed [34, 35]. In the present study, the accumulation of Na^+^ in the roots increased significantly in the early stage of salt stress, and potassium showed the opposite result. Under salt stress, the K^+^ content increased with time, and the Na^+^/K^+^ ratio increased. High concentrations of Na^+^ can impair the ability of plants to accumulate essential nutrients [36], while K^+^ is required to maintain the stability and function of cell membranes and related enzymes. Maintaining sufficient K^+^ levels in plant tissues under salt stress depends on selective cell K^+^ and Na^+^ distribution [37]. Interestingly, although the amount of Na^+^ in young sugar beet roots increased, the increase in the Na^+^/K^+^ ratio compared to that of CK decreased at the later stage, which is likely responsible for the metabolite accumulation. Some studies have suggested that the metabolic regulation of osmotic pressure by the roots of Agrostis species can reduce Na^+^ toxicity and is the key to improving the salt tolerance of those species [38]. In addition, compared with wild-type plants, transgenic *Arabidopsis thaliana* plants under salt stress presented an increased AA accumulation and a reduced Na^+^/K^+^ ratio [39]. The salt tolerance of sugar beet is a complex trait that is determined by many physiological and metabolic pathways, including an increased content of compatible solutes [22]. Lv et al. believe that the most important factor for beet salt tolerance may be osmotic adjustment [40].

Our research attempts to reveal the self-protective mechanism of sugar beet in response to salt stress. After being subjected to salt stress, the plants exhibited significantly different adaptive responses to stress in the early and late stages of stress. On the basis of the morphological and physiological responses of sugar beet to salt stress, we carried out transcriptional and metabolomic studies and found significant alterations of the expression of key genes and the accumulation of metabolites involved in carbon and nitrogen metabolism in sugar beet under early (1 day) and long-term (7 days) salt stress.

### Flavonoids are correlated with salt stress

Using mass spectrometry, we found that flavonoids increased significantly under salt stress. Flavonoids are a major class of plant secondary metabolites that include strong antioxidants [41, 42], such as L-ascorbic acid (Asc), alpha-tocopherol (vitamin E) and apigenin. It is well known that intracellular flavonoids located in the plasma membrane, chloroplast, vacuole and nucleus are strongly induced by oxidative stress [43]. The expression of flavonoid pathway genes is closely related to the content of intracellular ROS. Indeed, our results indicated a three-fold increase in Asc content. In plants, Asc acts as a major redox buffer, a cofactor for enzymes, and a regulator of cell division and growth and participates in signal transduction [44, 45]. Khan et al. [46] reported that the properties of Asc and its various functions in plant growth and development are important for cell reduction, antioxidant capability, and regulation of various cellular mechanisms for defense against abiotic stress.

Apigenin 7-glucoside and luteolin have also been associated with stress in recent years. The study by Mostafa Hojati showed that chamomile (*Matricaria chamomilla* L.) is resistant to water stress, which is related to changes in apigenin-7-glucoside content [47]. The study have also shown that luteolin can regulate the accumulation of soluble substances in plants to resist salt stress and is related to nitrogen metabolism. In this experiment, a large amount of apigenin 7-glucoside accumulated in the early stage of stress, while luteolin accumulated in the late stage of stress, and the content of the plants under stress was 6 times that in the plants under the CK conditions. This indicates that the flavonoid metabolites produced by sugar beet might first respond to osmotic stress and then respond to ionic stress. Moreover, the KEGG annotation analysis revealed that the expression of a large number of genes related to phenylpropane biosynthesis is upregulated. Some studies have indicated that phenylpropane biosynthesis is related to flavonoid and lignin synthesis, which can improve the osmoregulatory effect of *Fraxinus mandshurica* [48]. Ma et al. reported similar conclusions with respect to buckwheat [49]. Therefore, the accumulation of Asc, apigenin-7-glucoside and luteolin may be involved in the salt response of plants.

### AAs are correlated with salt stress

When plants are subjected to salt stress, they can resist or alleviate the damage caused by the accumulation of small-molecule osmotic adjustment substances [50]. A previous study showed that the AA metabolism of barley is enhanced during salt stress, and the accumulation of small-molecule AAs helps to improve salt tolerance by improving osmotic regulation and by maintaining cell membrane stability [51]. Under 280 mmol/L NaCl treatment, the content of proline and free amino acids was higher in salt-tolerant variety T710MU than in salt-sensitive S710 [52]. Our experimental results show that the AA metabolism of sugar beet was significantly enhanced in the early stage of salt stress, resulting in significant accumulation of glutamine, asparagine, cysteine, citrulline and phenylalanine. Asparagine and cysteine, which are precursors of methionine and glutathione synthesis, can scavenge cytotoxins and protect protein SH groups from oxidation and ROS [53, 54]. Similarly, cysteine is a metabolic precursor of essential biomolecules such as vitamins, cofactors, antioxidants and many defense-related compounds [55]. In this study, the content of O-acetyl-L-serine also increased to some extent. The final step in the metabolism of cysteine is catalyzed by O-acetylserine (thiol) lyase (OASTL), which binds the reduced sulfur to O-acetylserine to produce cysteine; therefore, cysteine plays an important role in salt stress. Glutamate can be used to produce citrulline, proline and gamma-aminobutyric acid (GABA). Citrulline is reported to be a potent hydroxyl radical scavenger and a potent antioxidant that protects DNA and metabolic enzymes from oxidative damage [56–58]. Abiotic stress produces ROS, which induce the expression of anionic glutamate dehydrogenase (GDH) to form glutamate to synthesize proline in tobacco and grapevines [59]. This relationship may be the reason for the decrease in glutamate content at the beginning of stress.

Permeates, which support increased cell osmotic potential under salt stress, exist as many compounds, but their synthesis is not synchronized: glutamine, asparagine, cysteine and phenylalanine act at the onset of stress, while oxalate, betaine, allantoin and citrulline accumulate mainly in the late stage of stress. Other compatible solutes, such as sugars, appear to play only a minor role. In addition, these compatible compounds can be used to store nitrogen, and plants can use nitrogen when the osmotic pressure is reduced [60].

### OAs are correlated with salt stress

Consistent with changes in AAs in tissues, OAs (including cis-aconitic acid, 2-isopropylmalic acid, benzoic acid, alpha-ketoglutarate, and L-malic acid) increased in the roots at the beginning of salt stress. This pattern of variation may be related to the cation/anion disequilibrium degree, which accounts for a vital factor that determines the plant OA contents [61]. For plant that absorbs excessive cations in the roots, charge balance should be restored by more negative charges, which are offered via OA, including aconitate, citrate, malate and malonate [62, 63]. Therefore, the elevated OA levels detected within the roots could offset the charge imbalances [64]; alternatively, it might act as the solutions with metabolic activity to regulate osmotic pressure [65]. Our results showed that OAs play an important role on the first day of salt stress. Similar to that which occurred in the present study, Liu et al. [66] analyzed switchgrass under drought stress and found a large amount of AA and OA accumulation. Metabolomic analysis of buckwheat under salt stress also led to the same conclusion [49]. This suggests that plants may first rearrange OAs and AAs in response to salt stress [8].

### Sucrose metabolism is correlated with salt tolerance

In plants, sugars serve as metabolic resources and structural components of cells, and sugars also undergo osmotic adjustment under various stress conditions [67–69]. In our study, salt stress caused an increase in soluble sugar content, which is consistent with the results of the study by Wang et al. [22]. A previous study showed that exogenous glucose and sucrose contribute to the growth of triticale seedlings under salt stress [70]. In the present study, the carbon metabolism of sugar beet roots significantly increased in the early stage of stress, whereas the sucrose and lipid content decreased. Sucrose is the main source of carbon and energy for plant metabolism. Annunziata et al. [17] reported that the root system of durum wheat undergoes a rebalance of sucrose and nitrogen compounds in response to salt stress. SS [EC 2.4.1.13] and INV [EC 3.2.1.26] are considered key enzymes involved in sucrose metabolism. In this study, expression of the SS gene was upregulated after 1 day but did not change significantly after 7 days of salt treatment. However, the gene encoding INV was upregulated on both days. Moreover, the D-fructose-1 and 6P2 content increased under salt stress. These results indicate that sugar beet roots promote the decomposition of sucrose into other soluble sugars and enhance the activity of the TCA cycle under salt stress, satisfying the energy requirement for survival at the expense of slow growth, and this phenomenon becomes more obvious with increased duration of stress [71]. A previous study suggested that differences in tomato salt tolerance capability may be related to the ability to regulate both carbon allocation and sucrose metabolism [72]. The results of Mišić et al. provide strong evidence that the expression of cell wall INV in *Schenkia spicata* is regulated in a salt-dependent manner [73]. However, transcriptomic analysis of sea beet and cultivated beet showed that under acute salt stress, sugar metabolism, protein processing, transcriptional regulation, and signal transduction were unique to cultivated sugar beet [8]; thus, different beet varieties may require different treatment methods to relieve salt stress and different causes of such stress in different growth periods.

### Allantoin accumulation is related to salt tolerance

Comprehensive analyses of transcription and metabolism have revealed that allantoin is a very important metabolite. In the present study, betaine accumulation was 6.2 times greater in the plants at the end of salt stress than in the CK plants. Betaine is a well-known nitrogen-containing compound that enhances stress resistance and stabilizes both the quaternary structure of proteins and membranes against the adverse effects of drought, high salinity and extreme temperatures [74]. However, the accumulation of allantoin in the plants under salt stress was 6.9 times that of the plants under the CK conditions. Allantoin (5-ureidolactam or 5-ureidohydantoin) a compound that contained the heterocyclic nitrogen, is an intermediate during the metabolism of plant urea. Generally, the urea compounds are generated through purine catabolism, which exerts a vital part in the metabolism of plant nitrogen [75]. A growing number of studies have reported the accumulation of allantoin in plants as a response to various stress conditions; allantoin participates in plant stress responses and provides tolerance to abiotic stress factors [76–78]. Similar findings in chickpea under drought stress have been reported [79].

The results of this study indicate that the expression of the gene encoding XDH is upregulated. Similarly, studies in which XDH genes from grape were overexpressed in Arabidopsis have shown that allantoin accumulation activates the abscisic acid (ABA) signaling pathway, helps to remove ROS and plays an important role in the salt stress response [80]. Allantoin not only is used as a mobile nitrogen-rich compound but also protects plants from abiotic stress by reducing oxidative damage [78]. Watanabe et al. reported that the loss of ALN function can lead to a large accumulation of allantoin in *Arabidopsis thaliana*, increase the expression of stress-related genes and increase tolerance to drought stress and osmotic stress, and the authors reported that the specific role of allantoin is regulated via ABA [81]. Studies on Arabidopsis plants under salt stress have also shown that application of exogenous allantoin increased the stress tolerance of Arabidopsis seedlings [77]. The relevant analysis in our study revealed that most of the genes differentially expressed under salt stress were significantly related to allantoin, which indicates that allantoin is may be more important and effective than other metabolites in response to salt stress in sugar beet. In addition, the results of this study involve changes in two hormones, namely, melatonin and (S)-2-aminobutyric acid, which are likely to reflect new regulatory pathways and provide direction for subsequent research.

In summary, we speculate that sugar beet can rapidly undergo sugar metabolism in response to salt stress, enhance the TCA cycle, and then accumulate osmotic adjustment substances by the reconstitution of carbon and nitrogen metabolism. Among the osmotic adjustment substances, allantoin may be used in sugar beet roots or as a component for storing nitrogen under stress. The allantoin gene can be used as a key gene for subsequent research.

## Conclusion

In this study, we analyzed the adaptation mechanism of sugar beet to short- and long-term salt stress through transcriptomics and metabolomics approaches. Studies have found that sugar beet is well adapted to salinity through regulating carbon and nitrogen metabolism, mainly involving the sucrose metabolic and purine metabolic pathways. Importantly, allantoin might be closely related to the adaptive response of sugar beet to salt stress. Our findings provide insights into beet salt tolerance and provide a valuable foundation for further improving plant stress resistance.

## Methods

### Plant growth conditions and stress treatments

The experiment was conducted in a plant growth chamber (23 °C/18 °C, day/night) at Northeast Agricultural University. Seeds of sugar beet (KWS0143, supplied by KWS company, Germany) were planted in pots that contained vermiculite and were maintained under a 14-h/10-h photoperiod with a light intensity of 450 μmol m^− 2^ s^− 1^ and a relative humidity of 60 ± 5%. After the seedlings emerged, each pot was watered with a half-strength Hoagland nutrient solution once daily for 10 days. Uniform seedlings were then transferred to separate 20-L plastic containers (length, 100 cm; width, 22 cm; height, 15 cm) that contained half-strength Hoagland nutrient solution and were continuously aerated with an air pump. The experimental design was completely randomized, consisting of one control and one salt treatment, with eight biological replications of each treatment. Each biological repetition of each treatment was performed in 4 independent containers. Salt treatments (NaCl and Na_2_SO_4_ at a 2:1 M ratio) started when the first pair of mature leaves fully developed. The concentration of Na^+^ was gradually increased from 100 mM to 300 mM in 3 days. A treatment that involved the addition of only nutrient solution was used as a blank CK. The pH of the nutrient solution was maintained between 7.0 and 7.2 with 2 N HCl or 2 N NaOH throughout the growth period. The nutrient solution was replaced every 2 days.

For each treatment, 8, 3 and 3 biological replicates were set up for metabolome profiling, transcriptome profiling and physiological parameter measurements, respectively. Samples were taken after 1 day and 7 days of salt stress. Half of the samples were immersed in liquid nitrogen and subsequently stored at − 80 °C for extraction of total RNA for transcriptomic and metabolomic analysis. The remaining samples were placed in an oven, dried at 105 °C for 15 min, and then dried at 80 °C for 2 days. The root/shoot ratio for each plant was calculated as the ratio of the plant’s dry root weight to its dry shoot weight.

### Analysis of RA and inorganic ion content

The RA (μg g^− 1^ h^− 1^) was measured according to the triphenyl tetrazolium chloride (TTC) method [82]. The dehydrogenase activity was considered an index of RA.

Dry root samples (0.1 g) were digested with HNO_3_/HClO_4_ (5/1 v/v) until the solution became clear. Na^+^ and K^+^ contents were determined via atomic absorption spectrophotometry (iCE 3500; Thermo Fisher Scientific, USA), and the inorganic ion contents were expressed in mg g^− 1^ DW.

### Determination of MDA, superoxide radical, H_2_O_2_ and proline contents

MDA was extracted with thiobarbituric acid (TBA), and the absorbance of the supernatant at 450, 532 and 600 nm was determined according to the method described by Shi et al. [83].

The superoxide anion (O_2_·^−^) content was determined according to the methods of Liu and Pang [84]. Potassium phosphate buffer (pH 7.8) was used to extract O_2_·^−^ from the plant materials via incubation at 25 °C for 20 min, the addition of both 17 mM sulfonamide and 7 mM alpha-naphthylamine, and incubation at 25 °C for 20 min. The absorbance at 530 nm was measured, and the O_2_·^−^ content was subsequently calculated via a standard curve.

The H_2_O_2_ content was determined according to the method of Velikova [85].After 0.1% (w/v) trichloroacetic acid was used to extract the root tissue, a phosphate buffer solution and 1 M KI were added. The reaction was terminated by incubation in the dark for 1 h, the absorbance at 390 nm was measured. Afterward, the H_2_O_2_ content was calculated via a standard curve, with the H_2_O_2_ content expressed in nmol g^− 1^ FW^− 1^.

The proline content was determined according to the methods of Bates [86]. Samples (0.5 g) were extracted with 3% (w/v) sulfated salicylic acid. Ninhydrin acid, glacial acetic acid, and the sample tissue were then mixed together and heated in a water bath at 100 °C for 1 h. The proline was extracted with toluene, the absorbance at 520 nm was measured, and the proline content was calculated via a standard curve, with the proline content expressed in μg g^− 1^ FW.

### Measurement of soluble sugar and protein contents

The soluble sugar content was determined according to the methods of Spiro [87]. One hundred microliters of the extract was added to a solution that consisted of 1.08 M H_2_SO_4_, 1.09 mM thiourea and 2.1 mM anthrone with the final volume being 3 ml. The mixture was then heated at 100 °C for 10 min. A calibration curve for D-glucose was established as a standard. The total protein content was determined via Bradford method [88].

### Analysis of antioxidant enzymes

The SOD activity was measured via the approach of Stewart and Bewley [89]; the enzymatic activity was expressed in units g^− 1^ (FW). One unit of SOD activity was defined as the amount of enzyme required for 1 mg of tissue proteins in 1 ml of reaction mixture to achieve an SOD inhibition rate of 50%. The POD activity was assayed by monitoring the formation of guaiacol at 470 nm according to the method of Fu [90]; the enzymatic activity was expressed in millimoles of guaiacol min^− 1^ g^− 1^ (FW). The CAT enzymatic activity was calculated via the system reported by Aebi [91].

### Metabolite profiling and data analysis

Samples were collected and thawed on ice; later, the 50% methanol solution was used to extract the metabolites. In brief, 120 μL of the 50% methanol solution cooled before hand was used to extract 20 μL sample under 1 min of vortexing, followed by 10 min of incubation under ambient temperature. Later, the mixture obtained was preserved under the temperature of − 20 °C. Afterwards, the mixture was centrifuged for 20 min at 4000 g to collect supernatants, which were later transferred to the new 96-well plates. The samples were stored at − 80 °C prior to LC-MS analysis.

All samples were analyzed by the LC-MS system following the machine operational procedures. Firstly, the UPLC system (SCIEX, UK) was employed for chromatographic separation. Additionally, reverse-phase separation was carried out on the ACQUITY UPLC BEH Amide column (100 mm*2.1 mm, 1.7 μm, Waters, UK). The temperature of column oven was kept to be 35 °C, with solvent A (25 mM NH_4_H_2_O+ 25 mM ammonium acetate) and solvent B (IPA:ACN = 9:1 + 0.1% formic acid) as the mobile phase, at the flow rate of 0.4 ml/min. The gradient elution conditions were set as follows: 0–0.5 min, 95% B; 0.5–9.5 min, 95 to 65% B; 9.5~10.5 min, 65%~ 40% B; 10.5–12 min, 40% B; 12–12.2 min, 40–95%B; and 12.2–15 min, 95% B [92]. The injection volume for each sample was 4 μl.

The metabolites eluted based on the column were detected using the Triple TOF 5600 plus high-resolution tandem mass spectrometer (SCIEX, UK), and Q-TOF operated under the negative and positive ion modes [92]. The ion source gases 1 and 2 were both set at 60 psi, while the curtain gas was set at 30 psi, and temperature of interface heater was set at 650 °C. With regard to the positive ion mode, its voltage floating of ion spray was 5000 V, while that was − 4500 V for negative ion mode. In addition, for evaluating the LC-MS system stability throughout the entire acquisition process, one sample for quality control (pooled based on all samples) was examined at an interval of 10 samples.

The raw data from the mass spectrometer were converted to readable mzXML data via ProteoWizard MSConvert software. Peak extraction was performed by XCMS software, and peak extraction QC was also performed. The extracted material was subjected to additive ion annotation by a camera and then subjected to primary identification via metaX software. The mass spectrometry primary information was used to identify and match the secondary information of the mass spectra with an in-house standard database. The identified candidate substances were subsequently annotated with metabolites via the Human Metabolome Database (HMDB), KEGG database, and other databases to explain the physicochemical properties and biological functions of the metabolites. The differentially accumulated metabolites were quantified and then screened via metaX software.

### Transcriptome RNA-seq process

Total RNA was extracted via a TRK1001 Total RNA Purification Kit (LC Science, Houston, TX) according to the manufacturer’s protocol [93]. The total RNA amount and purity were determined using a Bioanalyzer 2100 and RNA 6000 Nano LabChip Kit (Agilent, CA, USA), with RNA values > 7.0. After the total RNA was quantified, the eukaryotic mRNA was enriched by attachment to Oligo (dT) magnetic beads. The extracted mRNA was randomly broken into short fragments by fragmentation buffer, and the fragmented mRNA was used as a template to synthesize a strand of cDNA with six-base random primers (random hexamers), double-stranded cDNA synthesis in buffer, dNTPs, RNaseH and DNA Polymerase I [94]. AMPure XP beads were used to purify the double-stranded product, both T4 DNA polymerase and Klenow DNA polymerase were used to repair attachment of the sticky end of the DNA to the blunt end, A bases and linkers were added to the 3′ end, AMPureXP beads were used for fragment selection, and PCR amplification was ultimately performed to increase the final sequencing library. After the library was quantified, it was generated by an Illumina HiSeq 4000 instrument, and the sequencing read length was double-ended 2*150 bp (PE150) [93]; low-quality reads were removed. The raw sequence data are available under GEO Series accession number GSE114968. Differential expression and functional analysis of the genes were performed. The determination of the different gene expression levels was based on the above data analysis program, with HISAT software used to compare the sequencing data to the NCBI (https://www.ncbi.nlm.nih.gov/genome/?term=Beta+vulgaris) sugar beet reference genome. The transcripts were assembled using multiple alignments. Finally, R was used to graphically display the data results generated by Ballgown. Functional analysis of the DEGs included the use of GOseq for GO enrichment analysis and KOBAS for KEGG signaling pathway enrichment analysis. The expression levels of all transcripts were calculated by StringTie and Ballgown. StringTie was also used to determine mRNA expression levels by calculating the fragments per kilobase of transcript per million mapped reads (FPKM) [94]. Differentially expressed mRNAs and genes were selected by the R package Ballgown according to the criteria of log 2 (fold change) > 1 or log 2 (fold change) < − 1 and were statistically significant when *P* < 0.05 [95].

### Real-time PCR confirmation of salt-responsive genes

The expression levels determined by RNA-seq were verified by quantifying the expression of 18 randomly selected DEGs via qRT-PCR. The total RNA from the same batch as that used for RNA-seq was reverse transcribed into cDNA and used to verify the mRNA expression accuracy. qRT-PCR was performed on an ABI StepOnePlus real-time PCR instrument in conjunction with an SG Fast qPCR Master Mix Kit. The primers used were designed by Primer Express 5.0. The real-time reaction system consisted of 10.0 μL of SYBR Green qPCR Master Mix, 0.4 μL (10 μM) of each positive reaction primer, 7.2 μL of ddH_2_O, and 2.0 μL of cDNA. The amplification procedure consisted of 95 °C for 3 min; 45 cycles of 95 °C for 7 s; 57 °C for 10 s; 72 °C for 15 s; and then 72 °C for 10 min. The specificity of the primer pair was checked by sequencing the PCR product. The experiment was performed in triplicate, and the resulting melting curve was used to determine the specificity of the amplified fragment. Expression levels were calculated via the 2^-ΔCt^ or 2^-ΔΔCt^ method, and the data were analyzed by the Opticon Monitor Analysis Software 3.1 tool [96]. All the primers used are listed in Table S8.

### Statistical analysis

Data are represented as the mean of three or eight biological replicates ± the standard deviation (SD). The metabolomics analyses had eight replicates, and the other analyses had 3 replicates. Statistical significance was calculated via the Ballgown package of R 3.2.5 (R Core Team, Vienna, Austria). Both t tests and ANOVA were performed by SPSS Statistics 22.0 software (IBM, Chicago, IL). Tables and figures were prepared using Microsoft Excel 2013, GraphPad prism 8.3 software (San Diego, CA, USA) and R 3.2.2. Differences at *P* < 0.05 and 0.01 were considered significant and highly significant, respectively.

## Supplementary information

**Additional file 1: Table S1.** Raw metabolomic data at different stages of salt stress (*n* = 8). **Table S2.** Summary of the fold changes of the metabolomic data at different stages of salt stress. **Table S3.** Genes used for validation of sequencing (Seq) data by qRT-PCR. **Table S4.** The GO terms of DEGs from the two comparison groups (S_1vsCK_1 and S_7vsCK_7). **Table S5.** The KEGG pathway enrichment of DEGs from the two comparison groups (S_1vsCK_1 and S_7vsCK_7). **Table S6.** Important DEGs detected in the roots under salt stress (*P* < 0.05). Positive FC (fold change) numbers indicates the up-regulated expression, while negative FC indicates the down-regulated expression. **Table S7.** Description of DEGs associated with metabolites in sugar beet in response to salt. Positive FC (fold change) numbers indicates the up-regulated expression, while negative FC indicates the down-regulated expression. **Table S8.** List of primers of the genes for qRT-PCR.

## Data Availability

The raw RNA-seq data are available under GEO Series accession number GSE114968 (https://www.ncbi.nlm.nih.gov/geo/query/acc.cgi?acc=GSE114968). The datasets used and/or analyzed during the current study are available from the corresponding author upon reasonable request.

## Abbreviations

5-HIU: 5-hydroxyisourate
AA: Amino acid
ALN: Allantoinase
Asc: L-ascorbic acid
DEGs: Differentially expressed genes
ESI: Electrospray ionization
GABA: 4-aminobutyric acid
GDH: Glutamate dehydrogenase
H_2_O_2_: Hydrogen peroxide
LC-MS: Liquid chromatography-mass spectrometry
LEA: Late embryogenesis abundant
MDA: Malondialdehyde
O_2_·^−^: Superoxide anion
OA: Organic acid
OHCU: 2-oxo-4-hydroxy-4-carboxy-5-ureido-imidazoline
PC: Principal component
PC1: First principal component
PC2: Second principal component
ROS: Reactive oxygen species
TCA: Tricarboxylic acid cycle
TBA: Thiobarbituric acid
UPLC: Ultra-performance liquid chromatography
XDH: Xanthine dehydrogenase
